# Three cases of intra‐abdominal free air onset associated with COPD treated conservatively

**DOI:** 10.1002/ccr3.2687

**Published:** 2020-01-28

**Authors:** Saki Shuto, Megumi Naka, Chisato Konishi, Koichi Maekawa

**Affiliations:** ^1^ Department of Respiratory Medicine Ijinkai Takeda General Hospital Kyoto Japan

**Keywords:** chronic obstructive pulmonary disease, intra‐abdominal free air, pneumoperitoneum

## Abstract

Pneumoperitoneum is caused by respiratory disease in rare cases and can be treated conservatively. It is important to confirm physical abdominal examinations, laboratory data, and radiological findings to avoid unnecessary surgical procedures. The diagnosis of pneumoperitoneum associated with respiratory disease requires the exclusion of other fatal illnesses, especially gastrointestinal perforation.

## Graphical abstract

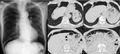

## 1 INTRODUCTION

Pneumoperitoneum is usually caused by gastrointestinal perforation and requires urgent surgical management. However, in rare cases, pneumoperitoneum is caused by respiratory disease such as chronic obstructive pulmonary disease (COPD) or asthma and it can be treated conservatively. We report three cases of intra‐abdominal free air associated with COPD that were treated conservatively.

## 2 CASE REPORT

### 2.1 Case 1

**Figure 1:**
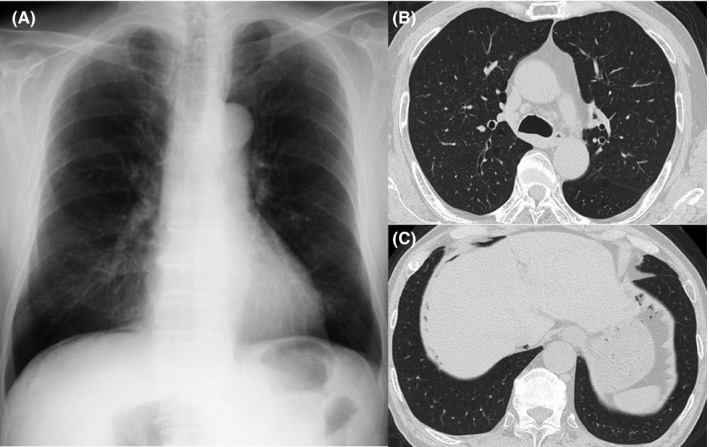
Radiological examination of Case 1. A, Chest X‐ray showed free air beneath the right diaphragm. B, C, Computed tomography showed mild pulmonary emphysema and intra‐abdominal air around the liver surface

A 75‐year‐old man had a regular visit for COPD (GOLD [Global initiative for chronic obstructive lung disease] stage 1) and annual X‐ray examination in May 2012. The chest X‐ray showed intra‐abdominal air beneath his right diaphragm (Figure 1A). He had quit smoking at the age of 74 and he was treated by inhaled bronchodilator and not using positive‐pressure ventilation nor long‐term oxygen therapy. He had no dyspnea or stomachache. He was afebrile with stable vital signs. The physical abdominal examination revealed no symptoms of peritonitis and hematological tests did not reveal any elevated inflammatory reactions. Computed tomography (CT) confirmed mild pulmonary emphysema (Figure 1B) and intra‐abdominal air around the liver surface (Figure 1C). Distinct gastrointestinal perforation, intraperitoneal inflammation, or mediastinal emphysema were not found. We considered the possibility of pneumoperitoneum associated with COPD and a digestive surgeon agreed that this could be managed conservatively. After 10 months, we confirmed the disappearance of his pneumoperitoneum.

### 2.2 Case 2

**Figure 2:**
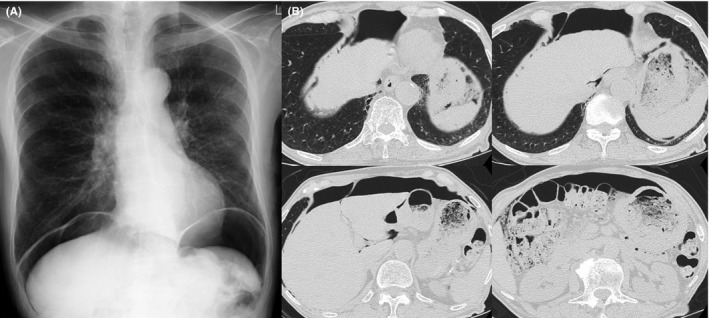
Radiological examination of Case 2. A, Chest X‐ray showed free air beneath both of the diaphragms. B, Computed tomography showed remarkable pulmonary emphysema and a large amount of intra‐abdominal air

**Figure 3:**
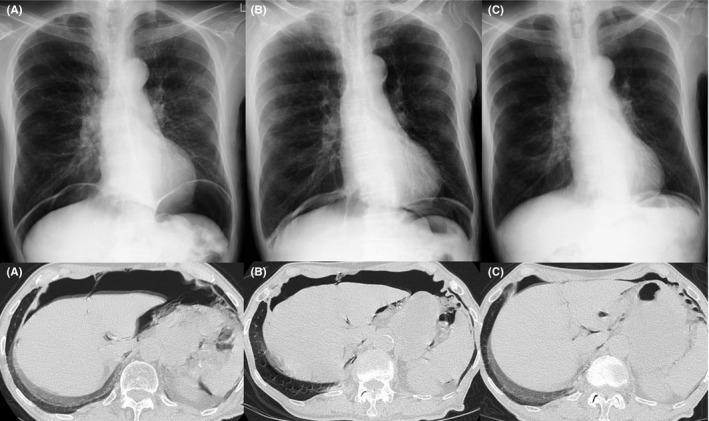
Radiological examination of Case 2. Chest X‐ray and computed tomography showed pneumoperitoneum spontaneously decreased. A, January 2014. B, March 2014. C, August 2014

An 87‐year‐old man had a regular visit for COPD (GOLD stage 4) and annual X‐ray examination in January 2014. The chest X‐ray showed intra‐abdominal air beneath both of his diaphragms (Figure 2A). He had a smoking history of 32 pack‐years and had quit smoking at the age of 50 and he was treated by inhaled bronchodilator and not using positive‐pressure ventilation nor long‐term oxygen therapy. He had no dyspnea or stomachache. He was afebrile with stable vital signs. The physical abdominal examination revealed no symptoms of peritonitis and hematological tests did not reveal any elevated inflammatory reactions. CT confirmed remarkable pulmonary emphysema and a large amount of intra‐abdominal air, which was not accompanied by distinct gastrointestinal perforation or intraperitoneal inflammation (Figure 2B). There was no obvious mediastinal emphysema. We again consulted a digestive surgeon and determined that the pneumoperitoneum was associated with COPD. He was treated conservatively until the pneumoperitoneum spontaneously decreased 7 months later (Figure 3).

### 2.3 Case 3

**Figure 4:**
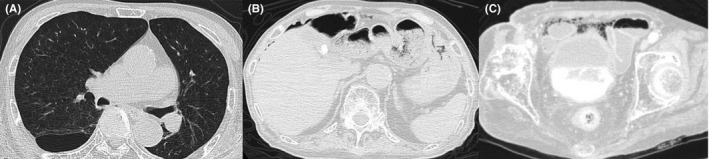
Radiological examination of Case 3. A, Computed tomography showed remarkable pulmonary emphysema. B, C, Computed tomography showed intra‐abdominal free air around the liver surface and in the pelvic cavity

An 89‐year‐old man with a history of COPD (GOLD stage 4) was taken to our hospital because of fever and dyspnea in December 2018. His temperature was 38.5°C and his SpO_2_ was 99% while inhaling 1 L/min oxygen. He had dementia, so the abdominal symptoms were difficult to evaluate. His hematological tests revealed a white cell count of 13 600/μL and C reactive protein level of 10.59 mg/dL. CT confirmed remarkable pulmonary emphysema (Figure 4A) and consolidation in the left lower lobe, which was consistent with pneumonia. There was no obvious mediastinal emphysema. Additionally, CT confirmed intra‐abdominal free air around the liver surface and in the pelvic cavity without other abdominal lesions (Figure 4BC). Because he had dementia and an elevated inflammatory reaction, it was difficult to decide whether he should undergo urgent surgical management. He was admitted to our hospital and an intravenous antimicrobial agent was administered to treat pneumonia. He inhaled 1 L/min oxygen but did not need artificial respirator including noninvasive ventilation. Though we considered his pneumoperitoneum to be associated with COPD, we carefully observed the pneumoperitoneum during fasting. As his SpO_2_ recovered and fever declined on day 3, he resumed oral intake. He had no subsequent worsening in abdominal symptoms and the pneumoperitoneum spontaneously decreased on day 11. He was discharged on day 14 of his hospital stay.

We demonstrate the patients' backgrounds in Table 1.

**Table 1:** Patient Characteristics

	Age	Sex	GOLD stage (%FEV_1_)	Emphysema Goddard score^13^	Pulmonary bullae	State of COPD	Treatment
Case 1	75	Male	Ⅰ (86.0%)	4	−	Stable	Conservative
Case 2	87	Male	Ⅳ (46.5%)	17	+	Stable	Conservative
Case 3	89	Male	Ⅳ (19.8%)	15	+	Exacerbated with pneumonia	Conservative

Abbreviations: COPD, chronic obstructive pulmonary disease; GOLD, global initiative for chronic obstructive lung disease.

## 3 DISCUSSION

Pneumoperitoneum without any symptoms of peritonitis, which is not caused by gastrointestinal perforation, can usually improve with conservative treatment. It is usually confirmed accidentally during a regular visit for a basal disease or medical checkup. The major etiologic mechanisms other than gastrointestinal perforation may be grouped under the following categories: thoracic, abdominal, gynecologic, and iatrogenic.^1^, ^2^ The thoracic causes include asthma, COPD, spontaneous rupture of pulmonary blebs, pneumonia, pneumothorax, and mediastinal emphysema. The abdominal causes include pneumatosis cystoides intestinalis, jejunal and sigmoid diverticulosis and aerophagia. The gynecologic causes include vaginal insufflation and pelvic inflammatory disease. The iatrogenic causes include laparotomy, endoscopic procedures, peritoneal dialysis, intermittent positive‐pressure ventilation, steroid, and immunosuppressive agents. All our cases are considered to result from thoracic causes (COPD) because others are absent. Pneumoperitoneum caused by respiratory disease is rare and we could find nine previously reported cases in the literature, including asthma,^3^, ^4^, ^5^, ^6^ COPD,^7^ bullous emphysema,^2^ pneumonia,^8^ interstitial pneumonia,^9^ and toxic fumes inhalation (Table 2).^10^

**Table 2:** Previous reported cases of pneumoperitoneum caused by respiratory disease

Age, Sex	Presenting complaint	Underlying pathology	Treatment	References
26, Male	Dyspnea, cough	Asthma	Conservative	Van der Klooster et al^3^
71, Male	Asthma exacerbation	Asthma	Conservative	Sekiya et al^4^
19, Male	Vomiting, epigastric pain	Asthma, peptic ulcer disease	Surgical ‐ no abnormality detected	Silbergleit et al^5^
20, Male	Asthma exacerbation	Asthma	Intubation, Nonsurgical	Lantsberg et al^6^
68, Male	Vomiting, abdominal pain	COPD	Conservative	Sturgeon et al^7^
54, Male	Abdominal pain	Bullous emphysema	Conservative	Mularski et al^2^
90, Male	Fever, dyspnea	Pneumonia	Surgical ‐ no abnormality detected	Sato et al^8^
77, Male	Nothing	Interstitial pneumonia	Conservative	Kochi et al^9^
23, Male	Dyspnea, cough	Toxic fumes inhalation	Conservative	Hillman^10^

Abbreviations: COPD, chronic obstructive pulmonary disease.

The onset mechanism of thoracogenic pneumoperitoneum is thought to be as follows. When respiratory tract internal pressure increases because of respiratory disease, air from ruptured alveolar walls or pulmonary blebs/bullas leaks into the stroma (including the perivascular sheath). The air reaches the mediastinum through the stroma and retroperitoneal cavity, then finally to the abdominal cavity.^7^, ^11^

All our cases had no obvious mediastinal emphysema, but we could estimate the passage of air from the CT scan of Case 2, which confirmed pulmonary emphysematous bulla in the right lower lobe and transmission of the air to posterior mediastinum and abdominal cavity (Figure 2B).

Through our cases, we showed that COPD could cause pneumoperitoneum in elderly patients. We confirmed that intra‐abdominal free air could also occur in the stable phase (Cases 1 and 2), not only during COPD exacerbation (Case 3). The degrees of obstructive ventilatory disturbance, emphysema, and pulmonary bulla ranged from mild to severe (Table 1). Mild COPD (GOLD stage 1) can develop thoracogenic pneumoperitoneum, so care should be taken when diagnosing the cause of pneumoperitoneum. However, we report a small number of patients. Further reports are needed to prove the association between pneumoperitoneum and backgrounds of patients who have COPD. There are also limitations to making a definitive diagnosis because our three cases could not have tests including barium meal or esophagogastroduodenoscopy to perfectly rule out abdominal pathology. However, we considered their pneumoperitoneum to be associated with COPD because they had no abdominal symptoms and their pneumoperitoneum improved conservatively without any surgical procedures.

When abdominal pain and distension are minimal and peritoneal signs, fever, and leukocytosis are absent, nonsurgical causes of pneumoperitoneum should be considered.^8^, ^12^ However, there exist many cases in which excluding the gastrointestinal perforation is difficult because half of the patients reported abdominal pain, elevated inflammatory reaction, and ascites. Actually, almost half of patients with pneumoperitoneum underwent unnecessary surgical procedures without evidence of gastrointestinal perforation.^2^ Considering that gastrointestinal perforation can be fatal, pneumoperitoneum patients should undergo surgery when gastrointestinal perforation cannot be excluded. When no findings suggest gastrointestinal perforation, it is important to avoid excessive invasion. Cases 1 and 2 could easily avoid unnecessary surgical procedures because they had no symptoms of peritonitis or elevated inflammatory reaction. However, Case 3 had dementia, the results of physical abdominal examination were unclear and he had an elevated inflammatory reaction, so it was difficult to decide whether he should undergo urgent surgical management. We should not easily determine that pneumoperitoneum associated with COPD. Careful observations and exclusion of gastrointestinal perforation are required when managing patients conservatively without surgical procedures.

## 4 CONCLUSION

We encountered three cases of pneumoperitoneum associated with COPD treated conservatively. It is important to confirm physical abdominal examinations, laboratory data, and radiological findings to avoid unnecessary surgical procedures.

## CONFLICT OF INTEREST

None declared.

## AUTHOR CONTRIBUTIONS

SS: involved in main work, data collection, and manuscript writing. MN: involved in data collection, manuscript revision. CK: involved in manuscript revision. KM: involved in final revision.
